# Thyroid Function and Cognition during Aging

**DOI:** 10.1155/2008/474868

**Published:** 2008-09-01

**Authors:** M. E. Bégin, M. F. Langlois, D. Lorrain, S. C. Cunnane

**Affiliations:** ^1^Research Center on Aging, Health and Social Services Centre, Sherbrooke Geriatrics University Institute, Sherbrooke, Quebec, Canada J1H 4C4; ^2^Endocrinology Service, Department of Medicine, Sherbrooke University, 3001 12th Avenue North, Sherbrooke, Quebec, Canada J1H 5N4; ^3^Department of Psychology, Sherbrooke University, 2500 University Boulevard, Sherbrooke, Quebec, Canada J1K 2R1

## Abstract

We summarize here the studies examining the association between thyroid function and cognitive performance from an aging perspective. The available data suggest that there may be a continuum in which cognitive dysfunction can result from increased or decreased concentrations of thyroid hormones. Clinical and subclinical hypothyroidism as well as hyperthyroidism in middle-aged and elderly adults are both associated with decreased cognitive functioning, especially memory, visuospatial organization, attention, and reaction time. Mild variations of thyroid function, even within normal limits, can have significant consequences for cognitive function in the elderly. Different cognitive deficits possibly related to thyroid failure do not necessarily follow a consistent pattern, and L-thyroxine treatment may not always completely restore normal functioning in patients with hypothyroidism. There is little or no consensus in the literature regarding how thyroid function is associated with cognitive performance in the elderly.

## 1. Introduction

Adequate thyroid function is essential for normal development and retention of cognitive function throughout life. The association between thyroid hormones and cognition has been recognized since the demonstration that cretinism stems from iodine and thyroid deficiencies. Low thyroid function at any age causes cognition to deteriorate because hypothyroidism prevents the brain from adequately sustaining the energy (glucose)-consuming processes needed for neurotransmission, memory, and other higher brain functions. Low brain uptake of glucose is commonly associated with deteriorating cognition and Alzheimer's disease and can be present decades before clinical evidence of Alzheimer's disease occurs [1, 2]. Brain hypometabolism therefore appears to be a precursor lesion increasing the risk of at least some forms of cognitive decline. Since thyroid hormone concentrations change with age and since cognitive decline is often concomitant with aging, physiological changes in thyroid function might be causally related to changes in cognition during normal aging.

Mental activities involved in the acquisition, storage, retrieval, and use of information are referred to here by the term “cognition” [3, 4]. Manifestations of cognitive behavior are achieved through the integration of a variety of processes and activities such as perception, imagery, memory, reasoning, problem solving, decision-making, and language. A summary of cognitive measurements used in thyroid function studies are shown in Table 1.

Thyroid function has been measured as serum concentrations of thyroid-stimulating hormone (TSH) or thyroid hormones (total or free thyroxine [T4], total or free tri-iodothyronine [T3], or T3 resin uptake). Most studies reported TSH, since it is very reproducible and is commonly believed to be the best marker of thyroid status, in combination with one or two thyroid hormones measurements.

Our aim here is to summarize the growing literature on the relationship between cognitive performance and thyroid function from an aging perspective. Several studies have been carried out to investigate whether thyroid function can explain the substantial individual differences in cognitive performance of older individuals. The characteristics of the association between thyroid function and cognition will be described first in healthy euthyroid individuals and then in patients with different degrees of hypothyroidism or hyperthyroidism.

## 2. Age-Related Changes in Cognition and Thyroid Function

Several changes in thyroid function occur during aging. Normal aging is associated with changes in thyroid hormone production and metabolism [5, 6]. Secretion of T4 and T3 is reduced in the healthy elderly (61–90 years old), but serum concentrations of total and free T4 remain relatively unchanged because T4 degradation is also reduced in the elderly. Circulating total and free T3 concentrations demonstrate a clear, age-dependent decline because of both reduced secretion and reduced peripheral conversion from T4. Serum reverse T3 (rT3) seems to increase with age. The decrease in serum T3 levels together with the increase in serum rT3 may indicate a decrease in peripheral hepatic metabolism of iodothyronines during aging.

Data on TSH changes during healthy aging are equivocal: some studies show unchanged TSH concentrations while others show mildly increased TSH in both men and women. After excluding subjects with subclinical hypothyroidism, the data suggest that TSH secretion may actually be slightly decreased in healthy elderly individuals [5]. A decline in both the circadian patterns and maximal nocturnal levels contributes in decreasing TSH levels in the elderly [5, 7].

The evaluation of normal thyroid function in the elderly is commonly confounded by subclinical hypothyroidism, by an increased prevalence of acute or chronic nonthyroidal illness, by drug use, and by nutritional deficiencies [6]. Some studies on healthy elderly subjects selected by strict criteria to exclude coexisting diseases suggest that an age-dependent decline in thyroid function is at least partially independent of nonthyroidal illnesses, particularly in the oldest old (>85 years) population [5, 8]. In the healthy elderly, thyroid function appears to be well preserved until the eighth decade of life, and a reduction of serum free T3 is only observed in the extremely old (100–110 years) [8].

An increased risk of declining cognitive function with aging is well-known, especially with regard to working memory, information processing speed, and long-term memory [4, 9–13]. Working memory is an essential cognitive resource whereby information is temporarily stored and made readily available to the various processes carrying on a specific task. Differences in cognitive efficacy can be related to discrepancies in working memory, including those presumably caused by normal or pathological aging [4]. Since it plays such a central role, alterations of working memory can have an effect on language processing, problem solving, decision-making, and encoding or retrieving of information in long-term memory.

Information processing speed is also an important resource, defined by the time parameters of a specific cognitive task. Shortest latencies are usually associated with youth and better performances. The label “cognitive slowing” has been applied to age-related increases in those latencies, which in turn can be held responsible for many aspects of declining cognitive functioning [4].

Efficiency of the three main aspects of long term memory—encoding, storage, and retrieval—declines over time [10]. A possible explanation could be that the elderly spontaneously deploy less effort at the time of exposure to properly encode information, thereby diminishing storage capacity and undermining subsequent retrieval attempts. This might be the reason why, for the elderly, retrieval tasks are especially difficult when summoned out of context [4]. An important decrease in mnesic performances has been observed, usually correlating with the complexity of the material, using either verbal or spatial stimuli, as well as declarative or procedural knowledge.

However, not all aspects of memory are equally affected with age [9]. For example, organization of long-term memory and priming of the information it contains remain relatively intact. Implicit inductive reasoning also seems to be well preserved, compared to explicit learning capacity. Broadly speaking, episodic memory, being the long-term memory concerned with items associated with a specific context in space and time, is generally more affected than semantic memory, which is the long-term memory concerned with general knowledge and values which have become quite independent of their acquisition context.

## 3. Cognitive Decline in Healthy Euthyroid Elderly

Table 2 shows relevant characteristics of five studies that sought to establish whether reduced thyroid function adversely affects cognitive function in healthy euthyroid individuals during aging. In a cross-sectional study involving a random sample of individuals aged from 49 to 71 years (mean age of 60 years), van Boxtel et al. [14] found that higher levels of TSH predicted lower levels of memory performance but that this association was not significant after correcting for mood status and the presence of possible thyroid dysfunction. Prinz et al. [15] suggested that total T4 levels and possibly free T4, but not total T3 nor TSH, were significantly associated with better cognitive performance in euthyroid men aged 65–78 years (mean 72 years) who had no overt physical or mental illness. In physically impaired 75–80-years-old euthyroid women, Volpato et al. [16] demonstrated that lower total T4 levels, (still within the normal range), but not TSH, were associated with a greater risk of decline in components of episodic memory over a 3-year period. Women with serum total T4 concentrations of 4.5–6.5 *μ*g/dL had a twofold risk of cognitive decline compared to women with serum total T4 concentrations of 8.1–12.5 *μ*g/dL. No association was found between baseline TSH level and change in cognitive function. These results were not substantially modified after adjustment for potential confounders including age, race, level of education, medication use, and presence of nonthyroidal illnesses.

Conversely, from a cross-sectional study of euthyroid very old women and men 75–96 years old (mean age = 84 years), Wahlin et al. [17] reported that, within the normal range, TSH levels but not free T4 were positively associated with episodic memory performance. The effects were independent of age, educational level, and depressive mood symptoms. No significant effects of TSH were depicted on verbal fluency, short-term memory, perceptual-motor speed, or visuospatial functioning. From further examination of very old women (*n* = 39) and men (*n* = 6), respectively, Wahlin et al. [18] provided longitudinal evidence of the impact of normal TSH variations on cognitive functioning. Their analyses revealed that declining TSH levels within the normal range were accompanied by a parallel decline in verbal fluency and visuospatial abilities, predicting episodic memory deficits after six years follow-up. Baseline TSH levels did not predict future decline of cognitive functioning. Conversely, Gussekloo et al. [19] found that low-free T3, but not TSH or free T4, was associated with an accelerated decline in global cognitive function as measured by the MMSE in an unselected general population of 558 individuals aged 85 years, of whom 85% were euthyroid, 5% were subclinically hypothyroid, 7% were clinically hypothyroid, and 3% were hyperthyroid, both at baseline and after a follow-up of 3.7 ± 1.4 years.

Clearly, the results from these studies are disparate and conflicting as to which indicator of thyroid function is the most relevant marker of specific cognitive function, and which domain of cognitive functioning is primarily affected by thyroid hormonal variations. However, the over all findings suggest that, although thyroid hormones may have an impact on a variety of cognitive functions, only a link to certain memory functions has been so far highlighted. Differences in results may be due to differences in sample selection, age ranges, choice of cognitive tests, and to higher TSH and cognitive variability with increasing age and limited number of thyroid function indices. More prospective follow up studies on a well-characterized selected population of healthy elderly are needed with repeated assessment of multiple indicators of thyroid function, ample specific cognitive measurements, and adequate assessment of mood status.

## 4. Cognitive Impairment in Subclinical Hypothyroidism

Subclinical hypothyroidism is defined as an elevated TSH levels in the presence of normal circulating T4 and T3 concentrations plus the absence of features of clinical hypothyroidism [20, 21]. About one-third of subclinically hypothyroid patients present mild, nonspecific symptoms possibly pointing to a mild hypothyroidism [22, 23] or a subclinical hypothyroidism, the latter being the term most frequently used in the studies examined.

Subclinical hypothyroidism may be a predisposing factor for cognitive impairment. Table 3 shows the results of different studies of cognitive impairment in subclinical hypothyroidism in order of increasing age. Patients with subclinical hypothyroidism for a long time (16 ± 6.2 months) manifested impaired cognitive activity as evidenced by significantly longer P3 wave latency of event-related potentials compared to healthy individuals [23]. Five cross-sectional studies suggest that young adult patients with subclinical hypothyroidism had mild dysfunction in learning, memory, and selective attention. Monzani et al. [24] found memory deficits in a group of 13 women and 1 man (29 to 47 years old) with untreated subclinical hypothyroidism compared to 50 euthyroid control subjects. Similarly, Baldini et al. [25] found that 19 young and middle-aged women with subclinical hypothyroidism showed worse memory, but no differences in scores of mental control, attention, or visuospatial abilities compared with 17 euthyroid control women. del Ser Quijano et al. [26] identified slower reaction time, reduced verbal fluency, and impaired visual memory in a group of 15 young adults with subclinical hypothyroidism compared with a group of 15 euthyroid control subjects. Cook et al. [27] reported that elderly patients with subclinical hypothyroidism performed more poorly than euthyroid individuals on measures of verbal recall as well as on the Mini-Mental State Examination but working memory and processing speed were unaffected. A decrease in verbal fluency was noted in the study of Manciet et al. [28] in a population sample of 425 individuals (65–85 years old or older) comprising 89.7% euthyroid subjects, 4.2% with subclinical hypothyroidism, 1.9% with clinical hypothyroidism, and 4.2% with hyperthyroidism.

In contrast, three studies failed to find cognitive impairment associated with subclinical hypothyroidism. No differences were found by Osterweil et al. [32] between the 14 patients with subclinical hypothyroidism and 30 controls. Similarly, in a group of elderly subjects aged 65–92 years, Luboshitzky et al. [33] reported no significant differences in cognitive impairment measured by the Mini-Mental State Examination in 39 untreated subclinically hypothyroid patients compared to 570 euthyroid controls. Bono et al. [29] provided evidence that subclinical hypothyroidism in 36 women barely affected their cognitive status but may have caused an age-related impairment of attentive functions.

The possibility of reversing some aspects of the cognitive impairment associated with subclinical hypothyroidism has been demonstrated after treatment with L-thyroxine. Two single-case studies showed that L-thyroxine treatment was associated with reversal of the cognitive impairment in each case [34, 35]. In a larger study, Bono et al. [29] also obtained a slight but significant improvement of verbal fluency in 36 women with subclinical hypothyroidism after 6 months treatment with L-thyroxine but this showed no correlation with TSH changes after treatment. Three uncontrolled studies showed improvements in memory skills after 3 or 6 months of treatment with L-thyroxine [24, 25]. The comprehensive study of del Ser Quijano et al. [26] revealed that L-thyroxine treatment was associated with significant improvements on multiple cognitive measures including attention, memory, verbal fluency, and executive functions compared to control participants.

The effects of L-thyroxine treatment were also investigated in three small randomized, placebo-controlled trials. A modest but statistically significant improvement in a composite memory score was obtained in patients treated with L-thyroxine for 10 months compared to placebo control patients [31]. However, the difference was small enough to be of questionable clinical importance. Compared to the placebo control group, Nyström et al. [30] reported that in a 6-month double-blind, crossover study using L-thyroxine treatment, up to 20% of patients improved their memory scores and reaction time but only half of them also improved their perceptual speed. Jensovsky et al. [23] found significant improvement of verbal, visual, and total memory scores after the normalization of TSH level by L-thyroxine treatment for 6 months in the treated individuals compared to the control individuals.

The interpretation of these studies is difficult due to variations in both the definition and the etiology of subclinical hypothyroidism, the presence or absence of symptoms, and to the limited number of subjects investigated. Nevertheless, taken together, the results suggest that evaluation and treatment of subclinical hypothyroidism in the elderly may be beneficial in view of its prevalence and its potential clinical importance. Indeed, subclinical hypothyroidism is common with estimates ranging from 5% to 17% in the general population, with an age-related increase to as many as 20% of women and 9.5% of men over the age of 60 years [6, 22, 33, 36–39]. The prevalence of subclinical hypothyroidism in men over the age of 74 years was 16% which approaches the 21% prevalence seen in women of the same age [40].

The elderly may be more vulnerable to the cognitive effects of subclinical hypothyroidism than young adults, suggesting that diagnosis and treatment may protect the aging brain from cognitive deterioration. However, it is not yet certain that favorable results obtained with middle-aged patients are predictive of favorable responses in elderly. Furthermore, different cognitive functions probably have varying sensitivity to hormonal or metabolic changes. Not all the cognitive defects related to thyroid failure are completely reversible with L-thyroxine therapy. Additional controlled studies are required to answer these questions.

## 5. Cognition Deficits in Clinical Hypothyroidism

Adult clinical hypothyroidism is the result of decreased serum levels of thyroid hormones and has a variety of adverse effects on cognitive function [32, 41–49]. Common cognitive deficits observed in middle-aged clinical hypothyroid patients include diminished general cognition, attention, learning, memory, and psychomotor speed (see Table 4). However, language comprehension and possibly sustained auditory attention are less impaired than other cognitive functions in clinical hypothyroidism. Whether or not there is a correlation between the cognitive alterations and the degree or the duration of hypothyroidism still needs to be clarified.

Although L-thyroxine successfully treats low thyroid status in the majority of hypothyroid patients, it is well recognized that a minority of patients have persistent symptoms. As shown in Table 4, several reports demonstrate that treatment of hypothyroidism during 3, 6, or 10.5 months still appears to be associated with only partial and inconsistent patterns of recovery of overall cognitive functioning. Similarly, the study of Wekking et al. [50] showed that cognitive defects, especially involving attention and verbal memory, persisted in a large group of hypothyroid patients, given sufficient L-thyroxine treatment over 5.5 years to normalize TSH. These results indicate that significant memory deficit in middle-aged adults with hypothyroidism can persist even after “adequate” thyroid replacement.

Three studies investigated the possibility that the brain becomes more susceptible to thyroid dysfunction in the seventh decade of life. Capet et al. [43] reported that the mean cognitive functions scores of treated patients or controls over 70 years old tended to be lower than those of treated patients or controls less than 60 years old. Osterweil et al. [32] compared cognitive performance of a group younger than 75 years to a group over 75 years. The older group performed worse on cognitive tests than the younger group, although there was no interaction between age and thyroid status. In a population with a prevalence of hypothyroidism of 14%, Luboshitzky et al. [33] reported that global cognitive impairment increased with age from 5.2% of the individuals aged 65–70 years to 35.5% in the age group of 85–90 years. Thus, age may increase vulnerability on cognitive tests to the effects of hypothyroidism.

## 6. Cognition Deficits and Hyperthyroidism

Hyperthyroid patients commonly present a variety of cognitive function deficits, although to a lesser degree than in hypothyroid patients. As shown in Table 5, the most common cognitive deficits observed in hyperthyroid patients include poorer performance on tests of attention, memory, mental alertness, and visuomotor speed [49, 51–54]. There are conflicting results concerning effects of hyperthyroidism on motor speed [55, 56]. Treatment of hyperthyroidism is followed by a partial return to baseline cognitive functioning [57].

The effects of experimentally raising thyroid hormones on cognitive performance have been reported in three studies. Increasing serum T3 levels to values within the hyperthyroid range by administrating 100 *μ*g T3 daily over 3 days to 14 young, euthyroid healthy men (20–37 years old) resulted in no significant change in either attention, memory, or visuomotor coordination, but a trend towards improved verbal fluency was seen [56]. Administration of supraphysiologic doses of L-thyroxine (500 *μ*g/d for 45 days on average) to 11 subjects (4 men, 7 women, mean age 37 years) did not change attention, memory, visuospatial organization, verbal learning, or verbal fluency [59]. In a placebo-controlled cross-over study in 24 healthy men, Münte et al. [58] found that administration of L-thyroxine in relatively high doses (300 *μ*g daily for 3 weeks) significantly improved visual processing. That cognitive functioning was little or not changed in these short-term studies suggests that the normal regulatory processes that modulate cognition, and which are disturbed in clinical hyperthyroidism, are not disrupted in healthy subjects by the use of supraphysiologic doses of thyroid hormones.

## 7. Thyroid Function and Alzheimer's Disease

Given the potentially increased risk of cognitive decline with thyroid dysfunction and that progressive cognitive decline is the central clinical feature of Alzheimer's disease, it is possible that thyroid status contributes, at least in part, to the clinical manifestation of Alzheimer's disease. Several clinical reports as well as laboratory and epidemiological studies support a link between thyroid hormones and Alzheimer's pathophysiology [41, 49, 60–71]. Studies examining the relationship between TSH and Alzheimer's disease have yielded contradictory results. Low serum TSH concentrations as well as high thyroxine levels were proposed as risk factors for Alzheimer's disease in some studies [72–74] but not in others [75, 76]. In a 6-year prospective follow-up study of mild cognitive impairment (which represents a transition between normal aging and Alzheimer's disease), Annerbo et al. [77] found a significant correlation between reduced TSH concentrations and patients converting from mild cognitive impairment to Alzheimer's disease. However, no significant correlation was found between TSH or free T4 levels and global cognition in euthyroid patients with Alzheimer's disease [78].

## 8. Discussion

Normal thyroid function appears to be an important factor in retaining optimal cognition in human aging [79]. Overall, many studies suggest that there may be a continuum characterizing the impact of thyroid function on cognition in which cognitive dysfunction results from either chronically increased or decreased concentrations of thyroid hormones. For example, Table 6 shows such continuum of specific cognitive defects with the degree of hypothyroidism. Even thyroid hormones and TSH levels within low-normal range appear to influence cognition performance, such that low-normal thyroid function appears associated with cognitive decline over time [16–18].

Many questions remain unresolved. For example, there is disagreement as to which of TSH, T4, or T3 is the best indicator of thyroid function, and best predictor of cognitive decline, especially in the elderly. The relationship between TSH and cognitive functioning appears to be inconsistent in euthyroid elderly [15–18]. Although it is the major modulator of thyroid hormone secretion, circulating TSH may not be a sufficiently sensitive or direct measure of thyroid hormone sufficiency for brain function. Moreover, serum TSH is influenced by a negative feedback loop associated with thyroid hormones and by the effect of other hormones and bioactive substances, such as cortisol, somatostatin, and cytokines [80]. TSH and cognitive performance may thus change in parallel, possibly due to a third variable. For example, TSH relationship with cognition may exist in a more specific or vulnerable population, in older, lower education, higher prevalence of cognition-related comorbidity.

Physiological studies provide support for a relationship between T4 and cognitive processing. The brain maintains levels of both T4 and T3 within a very narrow range, even during important fluctuations of circulating T4 [81]. Thus, possibly, even small changes in brain T4 concentrations could have consequences for cognitive function. Because brain function might be negatively affected by brain T4 in the lower range of normal, to function normally, the brain of older persons might have an increased need for or less efficient capacity to access or utilize T4. The basis for this shift could derive from aging-induced changes in T4 transport into specific brain regions, altered brain conversion of T4 to T3, or decreased brain thyroid receptor number or affinity.

An alternative explanation may be that the apparent paradox reflects differences in thyroid function before and after a threshold around the age of 80–85 years. Indeed, an age-dependent decline in thyroid function has been identified that is at least partially independent from associated nonthyroidal illnesses, particularly in those older than 85 years old [82, 83]. It is thus plausible that the correlation between cognitive decline may be stronger with T4 than with TSH in euthyroid individuals aged between 61 and 79 years but that a positive association with TSH appears with individuals older than 80 years old. This interpretation is supported by the studies reporting that increasing levels of TSH and decreasing levels of free T4, both representing lower thyroid function, were associated with a survival benefit [19, 82–84]. This metabolic change may reflect an adaptive mechanism to preventing excessive catabolism in the oldest elderly.

Our overall impression is that there is a substantial degree of disagreement regarding several aspects of the relationship between TSH, thyroid hormones, and cognitive performance. Most studies were adjusted for age, educational level, gender, and mood status, but differences in results that remain may still be due to differences in sample populations, exclusion criteria, age range, normal limits of TSH reference values, and choice of cognitive tests. Moreover, the interpretation of these results is complicated by the coexistence of age-related nonthyroidal illnesses which may contribute to serum thyroid hormone and TSH perturbations but for which adequate correction may not have been made [5, 85]. In turn, these thyroid indices may also affect cognitive performance by the degree and the duration of hypothyroidism [23, 32, 50], by practice effects [46, 57], by the use of insufficient thyroid function indices in most studies, by the wide individual variation in the rate at which TSH and cognitive functions deteriorate with increasing age [17, 18], by the effects of nutritional deficiencies [6], or by other endocrine perturbations, such as low sex steroids or high stress hormones, which could lead either directly or indirectly to cognitive dysfunction [18, 86].

No consensus has been reached about the health impact of subclinical thyroid dysfunction, for which different recommendations have been made about screening and treatment [20–22, 45, 54, 87, 88]. Different cognitive functions clearly have different sensitivity to hormonal or metabolic changes and not all the cognitive defects related to thyroid failure are completely reversible with thyroid replacement therapy.

Very few studies have examined the mechanism by which thyroid dysfunction might influence memory performance. There is some evidence that decreased cerebral blood flow in mild hypothyroidism occurs in regions mediating attention, motor speed, memory, and visuospatial processing [89]. High TSH concentrations may also decrease cerebral blood flow and glucose metabolism in clinical hypothyroidism [90]. However, comorbid cerebrovascular disease and elevated anticholinergic activity did not appear to be involved in memory impairment in elderly individuals with a mildly elevated serum TSH [27].

## Figures and Tables

**Table 1:** Cognitive domains and measures (AN: animal naming; CC: cube copying; CoS: Corsi's span; CST: concept shifting test; CVMT: continuous visual memory test; d2: d2 test; DRS: dementia rating scale; DS: digit spans forward and backward of WAIS-R; FMT: Milner facial memory test; FR: figure rotation from the Schaie-Thurstone adult mental abilities test; GNG: Go-NoGo; HT: Hooper test; IPALT: Inglis paired associates learning test; LDC: letter digit coding test; LMN: Luria m's and n's; LW: list of words; MHV: Mill Hill Vocabulary Scale; MMSE: Mini-Mental State Examination; MMMSE: Modified Mini-Mental State Examination; OR: oral reading; PASAT: paced auditory serial addition task; PM: Porteus maze; RBP: Rivermead behavioral profile; RCFT: Rey-Osterrieth complex figure test; RPM: raven progressive matrices test; RW: Rey's words immediate and delayed recall; ScT: scribble test; SDMT: symbol digit modalities test; SRT: selective reminding test (Buschke); ST: Stroop test; SVT: Shipley Vocabulary test; TMTA&B: trail making test A and B; WAIS: Wechsler adult intelligence scale; WAIS-R: Wechsler adult intelligence scale-revised; WD: word discrimination; WFT: word fluency test; WLT: word learning task; WMS: Wechsler memory scale; ZVT: Zahlenverbindungstest).

Cognitive domain	Measures
General intelligence	RPM, MHV, SVT, WAIS, WAIS-R, DRS
Attention and concentration	PASAT, TMTA&B, SDMT, ST, DS, CST, d2,
Memory	WMS, CVMT, FMT, RCFT, SRT, IPALT, DS, WLT, LW, RBP
Visuospatial organization	WAIS-R, CC, TMT(Part A), FR, HT, ScT, CoS
Language	WFT, AN, WD, RW, OR
Executive function	GNG, WFT, LMN, PM, TMT
Global cognition	MMSE, MMMSE
Cognitive or psychomotor speed	WAIS-R, TMT(Part A), WFT, LDC, ZVT

**Table 2:** Relation between thyroid function and cognition in euthyroid elderly (>65 years old) (A: attention; BD: block design of WAIS-R; CST: concept shifting test; DRS: dementia rating scale; DS: digit span forward and backward of WAIS-R; FR: figure rotation; FT3: free T3; G: global; LDC: letter digit coding test; LW: list of words; M: memory; MMSE: Mini-Mental State Examination; MRT: median reaction time tests; NS: not specified; NTI: nonthyroidal illnesses; O: orientation; PS: psychomotor speed; RBP: Rivermead behavioral profile; ST: Stroop test; TMTA&B: trail making test A and B; TSH: thyroid stimulating hormone; TT4: total T4; VF: verbal fluency; VSA: visuospatial ability; WAIS-R: Wechsler adult intelligence scale-revised; WLT: word learning task).

Source	Age, years		Gender			Population health status	Altered cognitive domain	Thyroid function indicator	Measures
	mean	range	women	men	N				
van Boxtel et al. [14]	60	49–71	NS	NS	120	Healthy + hypo	M	TSH	WLT, CST, ST
Prinz et al. [15]	72	65–78	0	44	44	Healthy	G, VF	TT4	WAIS-R, DRS, VF, FR, RBP, MMSE, MRT
Volpato et al. [16]	72	75–80	464	0	464	Heathy + NTI	O, M	TT4	MMSE
Wahlin et al. [17]	84	75–96	159	41	200	Healthy	M	TSH	DS-WAIS-R, LW, TMTA&B, BD
Wahlin et al. [18]	NS	75–93	39	6	45	Healthy	VF, VSA → M	TSH	RT, BD, VF, TMTA&B
Gussekloo et al. [19]		85–89	369	189	558	Mixed	G, A, M, PS	FT3	MMSE, ST, LDC, WLT

**Table 3:** Cognitive impairment in subclinical hypothyroidism (A: attention; BMT: Bingley's memory test; BVRT: Benton's visual retention test; CS: Corsi's span forward and backward; DC: drawing copy; DSB: digit span backward; DSCT: digit symbol coding test from the WAIS-III; DSF: digit span forward; DSS: digit symbol substitution; EF: executive functions; IFT: identical forms test of Thurstone; IST: Isaacs' set test of verbal fluency; LM: logical memory test; M: memory; MMSE: Mini-Mental State Examination; NBT: N back test; PS: perceptual speed; RAVLT: Rey auditory verbal learning test; RF: Rey's figure; RPM: Raven's progressive matrices; RT: reaction time; RW: Rey's words immediate and delayed recall; S: speed test; ScT: Scribble test; TMTAB: trail making test A and B; VF: verbal fluency; VF: verbal fluency test; WAIS: Wechsler adult intelligence scale; WFT: word fluency test; WLT: word learning test; WMS: Wechsler memory scale; ZBT: Zazzo's barring test).

Source	Participants				Impaired cognitive domain	Tests	Treatment outcome
	Age, years	Gender					
	Mean ± SD and/or (Range)	women	men	N			
del Ser Quijano et al. [26]	38 ± 9.5	12	3	15	A, M, VF, EF	MMSE, WAIS, RT, BVRT, RF, WLT, VF, TMTAB	Improvement after 6 months
(23–53)							
Monzani et al. [24]	39 ± 9	13	1	14	M	WMS	Improvement after 6 months
(29–47)							
Baldini et al. [25]	55 ± 9	19	0	19	M	WMS, ScT, S	Improvement after 3 months
(28–68)							
Bono et al. [29]	52 ± 13.5	36	0	36	A, VF	MMSE, DSF, DSB, CS, RW, TMTAB, RPM, DC, VF	Improvement after 6 months
	(31–70)						
Nyström et al. [30]	(51–73)	17	0	17	PS, M, S	IFT, BMT, RT	Improvement after 6 months
Jaeschke et al. [31]	68 ± 9.4	28	8	36	M, PS	LM, WLT, WFT, DSS, TMT	Improvement after 10 months
Jensovsky et al. [23]	62 ± 6.8	20	0	20	M	WMS	Improvement after 6 months
Manciet et al. [28]	(65–85)	256	169	425	VF	MMSE, BVRT, WAIS, ZBT, IST	No treatment
Cook et al. [27]	74 ± 3.9	NS	NS	15	M,VF	MMSE, RAVLT, DSCT, NBT, DSB	No treatment

**Table 4:** Cognitive deficits in adult clinical hypothyroidism (AN: animal naming; BD: block design of WAIS-R; BEC-96: recall and visuoverbal recognition test; CC: cube copy; COWA: controlled oral word association; CVLT: California verbal learning test; CVMT: continuous visual memory test; DRS: Dementia rating scale (Mattis); DS: digit symbol subtest; FMT: Milner facial memory test; GNG,: Go-No go; HT: Hooper test; IPAL: Inglis paired associates learning; LMN: Luria m's and n's; MHV: Mill hill vocabulary scale; MMSE: minimental sate examination; MMMSE: modified Mini-Mental State Examination; OAS: object assembly subsets of WAIS-R; OR: oral reading; PASAT: paced auditory serial addition task; PM: Porteus mazes; ROCF: Rey-Osterrieth complex figure test; RPM: Raven progressive matrices test; SDMT: symbol digit modalities test; SRT: selective reminding test (Buschke); SVT: Shipley vocabulary test; TMT(A): Trail making part A; TMT(A,B): Trail making test parts A and B; VLT: verbal learning test ; WAIS: Wechsler adult intelligence scale; WAIS-R: Wechsler adult intelligence scale-revised ; WD: word discrimination; WFT: word fluency test; WMS: Wechsler memory scale).

Cognitive domain	Source	Altered	Treatment outcome	Measures
General intelligence	Capet et al. [43]	Yes	Improvement after 6 months	WAIS-R
	Mennemeier et al. [46]	Yes	Not generally affected	WAIS-R
	Haggerty et al. [45]	Yes	No change after 3 months	WAIS, DRS
	Crown [44]	Yes	Improvement after 8 months	RPM, MHV, SVT

Attention	Miller et al. [47]	No	No improvement	TMT(A)
	Capet et al. [43]	Yes	Improvement after 6 months	TMT(A), WMS,
	Mennemeier et al. [46]	No	Improvement after 7 months	PASAT
	Osterweil et al. [32]	Yes	Improvement after 8 months	TMT(A), SDMT
	Whybrow et al. [49]	Yes	No change after 10.5 months	TMT(A,B)

Memory	Miller et al. [47]	Yes	Improvement after 3 months	WMS, CVLT, RCFT
	Burmeister et al. [42]	Yes	Improvement	VLT
	Capet et al. [43]	Yes	Improvement after 6 months	WMS, BEC-96, WMS,
	Mennemeier et al. [46]	Yes	No change (but treatment may arrest further decline)	CVMT, FMT, RCFT, SRT, WMS
	Osterweil et al. [32]	Yes	Improvement	IPAL
	Haggerty et al. [45]	Yes	Not treated	WMS

Visuospatial abilities	Capet et al. [43]	Yes	Improvement after 6 months	TMT(A), WAIS-R, HT
	Mennemeier et al. [46]	Yes	Improvement	WAIS-R (BD, OAS)
	Osterweil et al. [32]	Yes	No improvement	CC

Language (expression)	Miller et al. [47]	No	No change	AN
	Capet et al. [43]	No	No improvement after 6 months	WFT
	Mennemeier et al. [46]	No	Not treated	WFT
	Osterweil et al. [32]	Yes	No improvement	WFT (Animals)

Language (reception)	Osterweil et al. [32]	No	Not treated	WD, OR

Executive functions	Miller et al. [47]	No	No change	TMT(B), COWA
	Mennemeier et al. [46]	No	Not treated	GNG, WFT, LMN
	Whybrow et al. [49]	Yes	No change	PM

Psychomotor speed	Burmeister et al. [42]	No	No change	WAIS-R
	Capet et al. [43]	Yes	Improvement after 6 months	WAIS-R, WFT, TMT(A, B)

Global cognition	Capet et al. [43]	Yes	Improvement after 6 months	MMSE
	Osterweil et al. [32]	Yes	No improvement	MMMSE
	Peabody et al. [48]	Yes	Progressive decline	MMSE

**Table 5:** Cognitive changes in (natural and experimental) hyperthyroidism.

Cognitive domain	Hyperthyroidism			
	Natural	Experimental		
		T3^(b)^	T4^(d)^	T4^(e)^
		100 *μ*g/day	300 *μ*g/day	500 *μ*g/day
		for 3 days	for 21 days	for 45 days
Attention	Impaired^(a)^	No change	Not reported	No change
Memory	Impaired^(a)^	No change	Not reported	No change
Visuospatial abilities	Impaired^(a)^	No change	Impaired	No change
Motor speed	Impaired^(a),(b)^	Not reported	Not reported	Not reported
	No change^(c)^			
Verbal fluency	No change^(b)^	Accelerated	Not reported	No change

^(a)^Whybrow et al. [49], MacCrimmon et al. [51], Alvarez et al. [52], Trzepacz et al. [53], Toft [54]; ^(b)^Kathman et al. [56]; ^(c)^Zeitlhofer et al. [55]; ^(d)^Münte et al. [58]; ^(e)^Baethge et al. [59].

**Table 6:** Continuum of specific cognitive defects with the degree of hypothyroidism.

Hypothyroidism status	Attention	Memory	Speed	Verbal fluency	Visuospatial abilities
Low normal	0/1^(a)^ (0%)	5/6 (83%)	1/5 (20%)	1/5 (20%)	1/4 (25%)
Subclinical	2/4 (50%)	6/8 (75%)	2/5 (40%)	3/5 (60%)	0/3 (0%)
Clinical	3/5 (60%)	6/6 (100%)	1/2 (50%)	1/4 (25%)	3/3 (100%)

^(a)^Number of studies reporting cognitive defects over total number of studies.
